# The [(Bn-tpen)Fe^II^]^2+^ Complex as a Catalyst for the Oxidation of Cyclohexene and Limonene with Dioxygen

**DOI:** 10.3390/molecules29163755

**Published:** 2024-08-08

**Authors:** Katarzyna Rydel-Ciszek, Andrzej Sobkowiak

**Affiliations:** Department of Physical Chemistry, Faculty of Chemistry, Rzeszów University of Technology, Al. Powstańców Warszawy 6, 35-959 Rzeszów, Poland

**Keywords:** dioxygen activation, iron(II)-Bn-tpen complex, cyclohexene oxidation, limonene oxidation, pentadentate ligands

## Abstract

[(Bn-tpen)Fe^II^(MeCN)](ClO_4_)_2_, containing the pentadentate Bn-tpen–*N*-benzyl-*N*,*N′*,*N′*-tris(2-pyridylmethyl)-1,2-diaminoethane ligand, was studied in the oxygenation of cyclohexene and limonene using low-pressure dioxygen (0.2 atm air or 1 atm pure O_2_) in acetonitrile. 2-Cyclohexen-1-one and 2-cyclohexen-1-ol are the main products of cyclohexene oxidations, with cyclohexene oxide as a minor product. Limonene is oxidized to limonene oxide, carvone, and carveol. Other oxidation products such as perillaldehyde and perillyl alcohol are found in trace amounts. This catalyst is slightly less active than the previously reported [(N4Py)Fe^II^(MeCN)](ClO_4_)_2_ (N4Py–*N,N*-bis(2-pyridylmethyl)-*N*-(bis-2-pyridylmethyl)amine). Based on cyclic voltammetry experiments, it is postulated that [(Bn-tpen)Fe^IV^=O]^2+^ is the active species. The induction period of approx. 3 h during cyclohexene oxygenation is probably caused by deactivation of the reactive Fe(IV)=O species by the parent Fe(II) complex. Equimolar mixtures of Fe(II) salt and the ligand (in situ-formed catalyst) gave catalytic performance similar to that of the synthesized catalyst.

## 1. Introduction

The catalysis of hydrocarbon oxidation processes leading to industrially sound products remains a challenging problem in chemistry. Cyclohexene and limonene are abundant raw materials whose oxidation leads to valuable intermediates used in the polymer, pharmaceutical, food, and fragrance industries, as well as surfactant industries [1,2,3,4,5,6,7,8,9]. Molecular oxygen (dioxygen) is an attractive oxidant for both environmental and economic reasons.

**Scheme 1:**
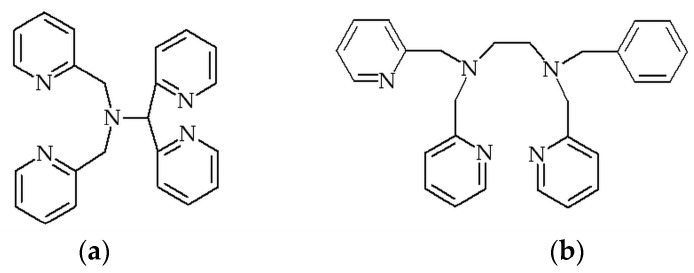
Structures of (**a**) N4Py and (**b**) Bn-tpen ligand.

**Scheme 2:**
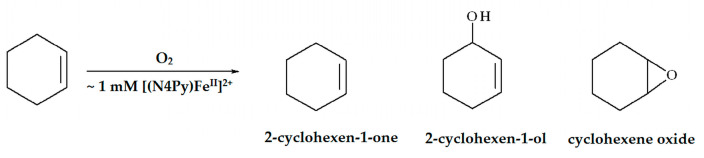
Products of cyclohexene oxidation with dioxygen catalyzed by [(N4Py)Fe^II^]^2+^.

In our recent article [1], we have reported that the [(N4Py)Fe^II^(MeCN)](ClO_4_)_2_ complex [N4Py–*N,N*-bis(2-pyridylmethyl)-*N*-(bis-2-pyridylmethyl)amine] (Scheme 1a) in acetonitrile (MeCN) catalyzes the oxidation of cyclohexene and limonene by dioxygen. Cyclohexene was oxidized mainly to 2-cyclohexen-1-one and 2-cyclohexen-1-ol; cyclohexene oxide was formed in much smaller amounts (Scheme 2).

**Scheme 3:**
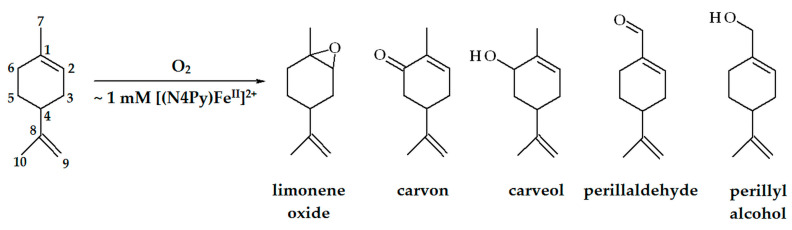
Products of limonene oxidation with dioxygen catalyzed by [(N4Py)Fe^II^]^2+^.

The oxidation of limonene led to limonene oxide, carvone, and carveol as the main products. Perillaldehyde and perillyl alcohol were also present in the products, but in trace amounts (Scheme 3).

We have postulated that the simultaneous combination of [(N4Py)Fe^II^]^2+^ dioxygen and substrate causes the formation of the iron(IV)-oxo adduct [(N4Py)Fe^IV^=O]^2+^, and the adduct initiates the catalytic cycle.

The [(Bn-tpen)Fe^II^(MeCN)](ClO_4_)_2_ complex [Bn-tpen–*N*-benzyl-*N*,*N′*,*N′*-tris(2-pyridylmethyl)-1,2-diaminoethane (Scheme 1b)] has been reported to have properties similar to those of the [(N4Py)Fe^II^]^2+^ complex. In reaction with iodosobenzene (PhIO), the [(Bn-tpen)Fe^II^]^2+^ complex also forms the iron(IV)-oxo adduct [10]. Both complexes have been intensively investigated, and a detailed discussion of their properties, also in relation to oxidation of organic substrates, is provided in our previous article [1]. In addition, the structures of both complexes and their adducts with oxygen as well as their physicochemical properties have been characterized in detail. Table 1 summarizes the papers presenting the properties of the complexes with both ligands.

The analysis of the data presented in the articles listed in Table 1 generally shows close similarities between the physicochemical properties of the complexes with the Bn-tpen and N4Py ligands. The same observations have been reported for the reactivity of the complexes. For example, the sulfoxidation of aryl 1-methyl-1-phenylethyl sulfides by iron(IV)-oxo complexes with both ligands occurs via an electron transfer−oxygen rebound mechanism and not through direct oxygen transfer [28]. Similarly, iron(II) complexes of both ligands are potent inhibitors of the 20S proteasome [29]. Iron(IV)-oxo complexes mimic flavone synthase enzymes that oxidize flavanones to flavones [30,31]. In the last case, the [(Bn-tpen)Fe^IV^=O]^2+^ complex is much more efficient, whereas in the previous cases, the differences between the complexes are not as strong.

The rate constants of electron transfer from a series of electron donors to various high-valent metal oxo complexes have been evaluated in light of Marcus theory to determine the reorganization energy for electron transfer [32]. The values for [(Bn-tpen)Fe^IV^=O]^2+^ and [(N4Py)Fe^IV^=O]^2+^ were the highest, with the value for the last complex approximately 0.2 eV higher. Based on EXAFS measurements, it has been reported [10] that in the series of complexes [*L*Fe^II^]^2+^, [*L*Fe^III^–OOBu*-t*]^2+^, and [*L*Fe^IV^=O]^2+^, for *L* = N4Py, five nitrogen atoms are located in the first coordination sphere of iron with a small range of Fe–N bond lengths. For *L* = Bn-tpen, greater variability in the length of Fe–N bonds is observed, and the first coordination sphere contains four nitrogen atoms; the same behavior was observed for these complexes with the tetradentate ligand TPA [TPA–*N,N,N*-tris(2-pyridylmethyl)amine]. On the other hand, it has been shown [33] that in the [(N4Py)Fe^II^]^2+^ complex, one nitrogen atom of the N4Py ligand can free the iron ion coordination site for a water molecule or hydrogen ion.

An interesting observation on the transformation of [(Bn-tpen)Fe^III^(OOH)]^2+^ in the presence of acids has been reported [34]. In acetonitrile, the addition of an acid with a value of p*K*_a_ greater than 8.5 causes homolysis of the O–O bond, producing an iron(IV)-oxo species and a hydroxyl radical, whereas in the presence of an acid with p*K*_a_ value less than 8.5, a highly reactive iron(V)-oxo species and a water molecule are formed through proton-assisted O–O bond heterolysis.

Taking into account the above considerations, we have decided to use the [(Bn-tpen)Fe^II^]^2+^ complex as a catalyst for the oxidation of cyclohexene and limonene with dioxygen. To the best of our knowledge, the complex has not been used in the activation of dioxygen for the oxidation of cyclohexene and limonene. Only peroxo and hydroperoxo complexes, [(Bn-tpen)Fe^III^–O_2_]^+^ and [(Bn-tpen)Fe^III^–OOH]^2+^, respectively, have been reported to oxidize cyclohexene [35].

## 2. Results and Discussion

### 2.1. Oxidation of Cyclohexene

When transition metal complexes are used as homogeneous catalysts in solution, the question usually arises of whether the application of the catalyst formed in situ by mixing stoichiometric amounts of ligand and a simple salt of metal is comparable to the use of the synthesized metal complex. Therefore, we have performed the oxidation of cyclohexene using both forms of the catalyst.

Cyclohexene is oxidized to ketone (2-cyclohexen-1-one), alcohol (2-cyclohexen-1-ol), and epoxide by dioxygen in the presence of [(Bn-tpen)Fe^II^]^2+^ as a catalyst. The concentrations of the products obtained after a 24 h reaction time in a continuous flow of dioxygen or air in the presence of different concentrations of substrate and synthesized catalyst are presented in Table 2. For example, using 1 M cyclohexene and 1 mM of the catalyst in the dioxygen atmosphere, 120 mM ketone, 50 mM alcohol, and 11 mM epoxide were obtained, which means that 0.18 M of the products were formed with 181 product/catalyst turnovers. The yield is slightly lower than reported for [(N4Py)Fe^II^]^2+^ catalysts under the same experimental conditions, where 114 mM ketone, 77 mM alcohol, and 10 mM epoxide were obtained, giving 201 turnovers [1]. However, the present system is slightly more selective. Generally, the amount of alcohol formed is less than half that of ketone. 

Relatively high concentrations of the substrate have been used to avoid further oxidation of the products formed. We have previously reported [36] that under the same experimental conditions, the oxidation of cyclohexene did not occur in the absence of a catalyst, and in the presence of uncomplexed iron(II), only traces of ketone and alcohol were detected. 

From the data presented in Table 2, it follows that there is no substantial difference in the reaction yields between the two forms of the catalyst used, i.e., synthesized (see Chemicals and Reagents) and formed in situ after mixing equimolar amounts of Bn-tpen and Fe(ClO_4_)_2_∙6H_2_O, especially for their lower concentrations. It is necessary to realize that by the use of hydrated iron salt, water is introduced to the system. For the highest concentration of catalyst investigated, taking into account the amount of water in the solvent (~0.02%), the total water content is approximately equal to 0.16%. Obviously, such amount of water does not influence the investigated process. This is in agreement with our previous finding that water content up to 1.1% in MeCN does not change the redox properties of the Fe^III^/Fe^II^ couple [37].

For low concentrations of the catalyst (0.5–2.5 mM), the product yields do not depend significantly on its concentration. The further increase in the catalyst concentration causes a decrease in the amounts of the products formed. The effect was observed in our previous studies on the activation of dioxygen by transition metal complexes and was caused by the deactivation of the catalysts used [1,38]. Another interesting observation is that the use of either dioxygen or air as an oxidant gives very similar amounts of the products.

**Figure 1:**
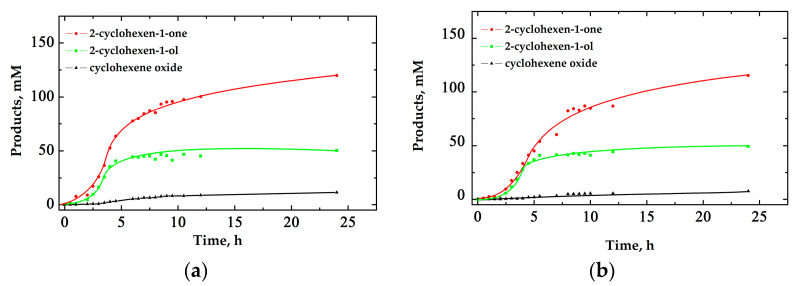
Product concentrations over time for the oxidation of 1 M cyclohexene with (**a**) dioxygen (p_O2_ = 1 atm) and (**b**) air (p_O2_ = 0.2 atm) in MeCN, in the presence of 1 mM synthesized [(Bn-tpen)Fe^II^]^2+^.

**Figure 2:**
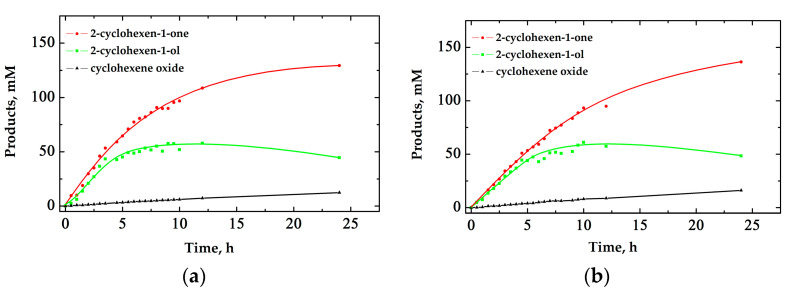
Product concentrations over time for the oxidation of 1 M cyclohexene with 10 mM (**a**) *t*-Bu–OOH and (**b**) HOOH in MeCN, in the presence of 1 mM synthesized [(Bn-tpen)Fe^II^]^2+^. The reaction was carried out in an air atmosphere (p_O2_ = 0.2 atm).

Figure 1 presents the dependence of the concentrations of the products on time for the oxidation of 1 M cyclohexene with dioxygen and air catalyzed by 1 mM synthesized [(Bn-tpen)Fe^II^]^2+^ in acetonitrile. For both dioxygen and air used as oxidants, an induction period can be observed in the product profiles. Similar behavior was observed when the catalyst prepared in situ was used under the same experimental conditions. The induction period has previously been observed during the oxidation of limonene by dioxygen catalyzed by the [(bpy)_2_Mn^II^]^2+^ (bpy–2,2′-bipyridine) complex in acetonitrile [38] but not for iron complexes. When 10 mM *tert*-butyl hydroperoxide (*t*-Bu–OOH) or hydrogen peroxide (HOOH) was introduced into the system at the beginning of the experiment, the induction period was not observed (Figure 2).

The product profiles indicate that the concentration of alcohol decreases slightly with time. This is caused by the further oxidation of the formed alcohol. In fact, oxidation of 50 mM of alcohol (the mean amount of alcohol formed after 24 h during cyclohexene oxidation) in the presence of 1 mM synthesized [(Bn-tpen)Fe^II^]^2+^ gives 5 mM and 4 mM of ketone after 24 h for dioxygen and air, respectively.

The existence of the induction period and the S-shaped product profiles could indicate that an autocatalytic mechanism occurs. It has been suggested that the presence of alcohol facilitates the formation of iron(IV)-oxo species during the autocatalytic oxidation of iron(II) [39]. In this case, in the presence of a hydrocarbon, a corresponding organic hydroperoxide should also be formed. However, during cyclohexene oxidation in the presence of 10 mM of 2-cyclohexen-1-ol, an induction period was also present (Figure S1). Furthermore, the introduction of triphenylphosphine before the analysis of the reaction mixture (Shul’pin test [40], also for alkenes [41]) only slightly increased the amount of alcohol (within experimental error), indicating that hydroperoxide does not play a substantial role in the reaction mechanism (Figures S2 and S3).

### 2.2. Oxidation of Limonene 

Because in the case of cyclohexene oxidation, the amounts of products obtained using synthesized and in situ forms of the catalyst do not differ substantially, the experiments for limonene oxidation were performed using only the catalyst formed in situ. [(Bn-tpen)Fe^II^]^2+^ formed in situ also catalyzes the oxidation of limonene by dioxygen. The main products are limonene oxide, carvone, and carveol. Perillaldehyde and perillyl alcohol are formed in trace amounts. Table 3 presents the product concentrations obtained after 24 h reaction time, using dioxygen and air as oxidants, for different concentrations of catalyst and substrate.

For all experimental conditions, the amount of the products formed, in decreasing order, was limonene oxide > carvone > carveol. For example, the combination of 1 mM catalyst and 1 M substrate in a dioxygen atmosphere after 24 h gave 62 mM limonene oxide, 38 mM carvone, 15 mM carveol, 2 mM perillaldehyde, and 2 mM perillyl alcohol, meaning that approximately 0.12 M substrate had reacted with 119 product/catalyst turnovers. The yield obtained was lower than when [(N4Py)Fe^II^]^2+^ was used as the catalyst. In this case, in the same experimental conditions, 0.18 M limonene reacted with 181 turnovers [1].

As follows from Table 3 for all catalyst concentrations investigated (0.5–10 mM), its concentration does not have a substantial influence on the amount of products formed. Generally, there were no significant differences in the concentrations of the products depending on the use of dioxygen or air as an oxidant. The amounts of products formed increased proportionally to the substrate concentration. 

**Figure 3:**
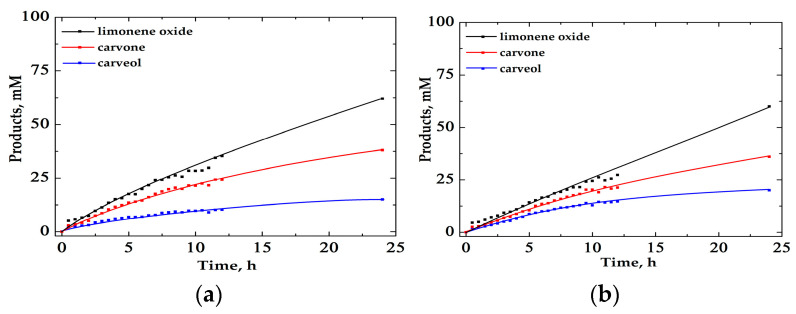
Product concentrations over time for the oxidation of 1 M limonene with (**a**) dioxygen (p_O2_ = 1 atm) and (**b**) air (p_O2_ = 0.2 atm) catalyzed by a 1 mM mixture of Bn-tpen and Fe(ClO_4_)_2_∙6H_2_O in MeCN.

From the time profiles of the product concentrations presented in Figure 3, it follows that the dependencies are almost linear up to 24 h reaction time. After this time, plateaus start to form for all products. The striking difference between the oxidation of cyclohexene and limonene under the same experimental conditions is the lack of an induction period for limonene.

### 2.3. Electrochemical Properties of the [(Bn-tpen)Fe^II^]^2+^ Complex

**Figure 4:**
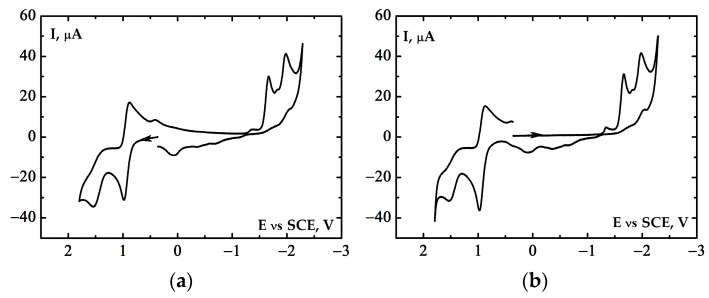
Cyclic voltammograms of 5 mM [(Bn-tpen)Fe^II^(MeCN)](ClO_4_)_2_ in MeCN containing 0.1 M (*t*-Bu)_4_NClO_4_ as a supporting electrolyte: (**a**) anodic scan, (**b**) cathodic scan performed first. Scan rate, 0.1 V/s, GCE (0.008 cm^2^, GCE—glassy carbon electrode), SCE vs. NHE +0.242 V (SCE—saturated calomel electrode, NHE—normal hydrogen electrode).

Cyclic voltammetry provides useful information on the interaction in the catalyst/substrate/oxidant system. Figure 4 illustrates the electrochemical behavior of the synthesized [(Bn-tpen)Fe^II^]^2+^ complex in acetonitrile in a wide potential window. 

Two oxidation peaks are observed at potentials +1.0 V and +1.5 V vs. SCE, which correspond to the oxidation of iron(II) to iron(III) and iron(III) to iron(IV) complexes, described by Equations (1) and (2), respectively.

[(Bn-tpen)Fe^II^]^2+^ − *e*^−^ → [(Bn-tpen)Fe^III^]^3+^ (1)

[(Bn-tpen)Fe^III^]^3+^ − *e*^−^ → [(Bn-tpen)Fe^IV^]^4+^ (2)

During the anodic scan, a very large oxidation peak was observed at a potential equal to +2.4 V (Figure S4). The origin of the peak is hard to interpret. It may have been be caused by oxidation of the components of the system and the products of their interactions. In view of the results of the paper, the peak will not be considered. Similarly, two cathodic peaks, probably caused by the ligand-centered reduction of the [(Bn-tpen)Fe^II^]^2+^ complex, are beyond the scope of the present research.

**Figure 5:**
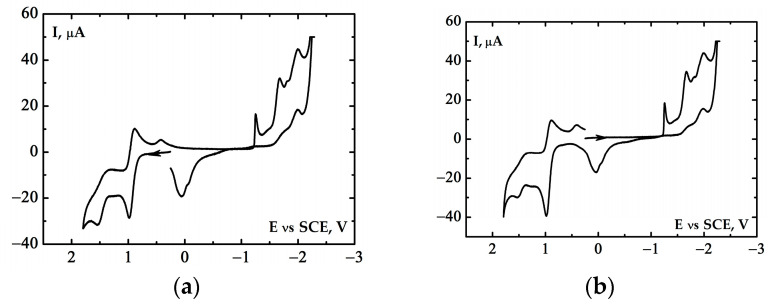
Cyclic voltammograms of a 5 mM mixture of Bn-tpen and Fe(ClO_4_)_2_∙6H_2_O in MeCN containing 0.1 M (*t*-Bu)_4_NClO_4_ as a supporting electrolyte: (**a**) anodic scan, (**b**) cathodic scan performed first. Scan rate 0.1 V/s, GCE (0.008 cm^2^), SCE vs. NHE +0.242 V.

Figure 5 presents a cyclic voltammogram of [(Bn-tpen)Fe^II^]^2+^ complexes prepared in situ by mixing 5 mM Fe(ClO_4_)_2_∙6H_2_O and 5 mM Bn-tpen. The shape of the voltammogram is analogous to that obtained for the synthesized one except for a small sharp peak in the cathodic region at approximately −1.3 V. This fact indicates that the [(Bn-tpen)Fe^II^]^2+^ complex is formed under these conditions and explains the similarity of the concentrations of products formed in the oxidation of cyclohexene by dioxygen by the use of both forms of the catalyst.

The first anodic peak at +1.0 V is reversible, whereas the second at +1.5 V is irreversible, which indicates that [(Bn-tpen)Fe^III^]^3+^ is stable under experimental conditions and [(Bn-tpen)Fe^IV^]^4+^ is not. Both oxidation processes (Equations (1) and (2)) are diffusion-controlled, which is confirmed by the linear dependencies of *I = f(ν*^1/2^*)*, where *I* is the value of a maximum peak current and ν is the potential scan rate [42] (see Figures S5 and S6).

**Figure 6:**
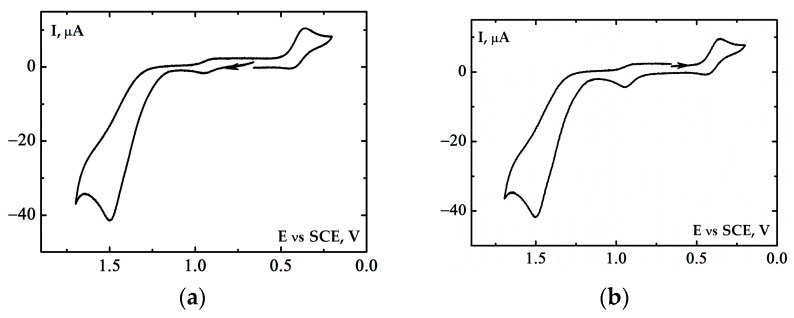
Cyclic voltammograms of the mixture of 5 mM Fe(ClO_4_)_3_ and 5 mM Bn-tpen in MeCN with 0.1 M (*t*-Bu)_4_NClO_4_: (**a**) anodic scan, (**b**) cathodic scan performed first. Scan rate 0.1 V/s, GCE (0.008 cm^2^), SCE vs. NHE +0.242 V.

In contrast to N4Py [1], Bn-tpen does not reduce Fe^3+^ ion. Figure 6 presents the cyclic voltammogram measured in the solution obtained by mixing 5 mM Fe(ClO_4_)_3_ and 5 mM Bn-tpen in MeCN. In the first anodic scan (Figure 6a), the iron(II) oxidation peak is almost not visible, whereas the iron(III) oxidation peak is well developed. However, in the first cathodic scan, the reduction peak originated from the reduction of [(Bn-tpen)Fe^III^]^3+^, which should be visible at +0.9 V as that generated by the oxidation of [(Bn-tpen)Fe^II^]^2+^, is not present. Instead, the first reduction peak appears at +0.35 V, probably as a result of the reduction of an iron-hydroxo complex due to the traces of water present in the investigated system. The tendency of iron(III) to form hydroxo complexes is well known [43,44].

**Figure 7:**
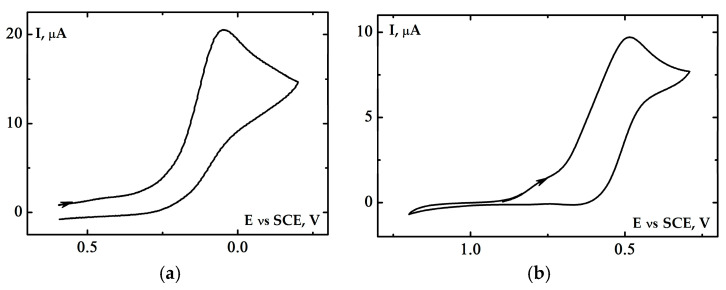
Cyclic voltammograms of (**a**) the mixture of 5 mM [(Bn-tpen)Fe^II^]^2+^ and 10 mM PhIO in MeCN [0.1 M (*t*-Bu)_4_NClO_4_] and (**b**) the same solution after the addition of 15 mM HClO_4_. Scan rate 0.1 V/s, GCE (0.008 cm^2^), SCE vs. NHE +0.242 V.

[(Bn-tpen)Fe^II^]^2+^ reacts with iodosobenzene (PhIO) to form [(Bn-tpen)Fe^IV^=O]^2+^ [10,17,19,21]. Figure 7a presents a cyclic voltammogram registered in a solution of [(Bn-tpen)Fe^II^]^2+^ and PhIO. The cathodic peak at +0.1 V corresponds to the reduction of [(Bn-tpen)Fe^IV^=O]^2+^. The process is diffusion-controlled (Figure S7). As indicated in [1] PhIO is not electroactive in the potential region. The addition of a proton source to the system results in the shift of the [(Bn-tpen)Fe^IV^=O]^2+^ reduction peak towards more positive values (+0.5 V, Figure 7b), which is characteristic of iron(IV)-oxo species [45].

**Figure 8:**
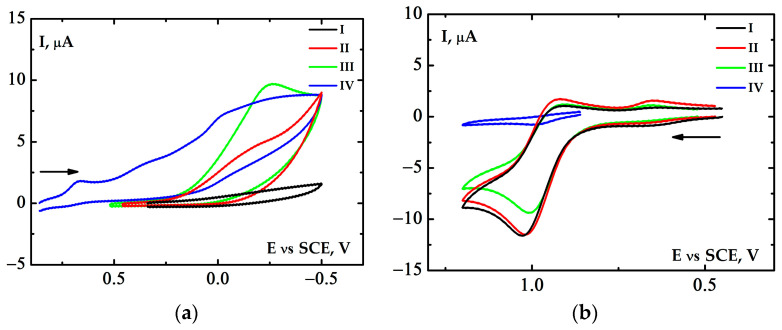
Cyclic voltammograms of 5 mM [(Bn-tpen)Fe^II^]^2+^ in MeCN with 0.1 M (*t*-Bu)_4_NClO_4_: (**a**) cathodic scan, (**b**) anodic scan performed first. (I)—in an Ar atmosphere, (II)—in a dioxygen atmosphere, (III)—as (II) after the addition of 0.5 M cyclohexene, (IV)—as (II) after the addition of 0.5 M limonene. Scan rate 0.1 V/s, GCE (0.008 cm^2^), SCE vs. NHE +0.242 V.

The cyclic voltammogram of [(Bn-tpen)Fe^II^]^2+^ in the presence of dioxygen shows a broad reduction peak located in the potential range of +0.1 V to −0.2 V (Figure 8a). It may suggest that the iron(IV)-oxo complex is formed in the system. However, the formation of iron(III)-hydroxo complexes cannot be excluded; their presence probably causes the broad nature of the observed reduction peak. The addition of cyclohexene or limonene induces the increase in the peak; in the presence of cyclohexene, it is better developed and shifted to −0.2 V. It would suggest that the presence of a substrate facilitates the formation of iron(IV)-oxo species. The oxidation peak of [(Bn-tpen)Fe^II^]^2+^ is barely altered by the presence of dioxygen. However, the addition of 0.5 M limonene causes the oxidation peak to practically disappear, while the addition of the same amount of cyclohexene results in a slight decrease in the height of this peak (Figure 8b).

These facts can be used to explain the observed induction period during the oxidation of cyclohexene. The presence of the [(Bn-tpen)Fe^II^]^2+^ complex in the system can deactivate the iron(IV)-oxo complex due to the formation of the diiron µ-oxo complex as presented in Equation (3) [46].

[(Bn-tpen)Fe^IV^=O]^2+^ + [(Bn-tpen)Fe^II^]^2+^ → [(Bn-tpen)Fe^III^–O–Fe^III^(Bn-tpen)]^4+^ (3)

Usually, µ-oxo iron complexes are considered less active in activating dioxygen for oxidation of organic substrates compared to monomeric species [47]. The formation of oxidation products after the induction period can be explained by the assumption of the reduction of the µ-oxo species to an iron(II) complex by cyclohexene, which is apparently a slow process. However, it was also reported that µ-oxo iron complexes catalyze the oxidation of organic compounds by hydroperoxides [48]. This fact can explain the lack of an induction period during cyclohexene oxidation when hydrogen peroxide or *tert*-butyl peroxide is introduced into the reaction system. The oxidation of the iron(II) complex in the presence of limonene and dioxygen prevents the decomposition of active iron(IV)-oxo species and therefore enables the oxidation reaction to occur immediately after mixing the reagents. These assumptions also seem to be confirmed by the fact that, for limonene oxidation, the TON for 10 mM of the catalyst is approximately fourth as much as in the case of cyclohexene oxidation (Table 2 and Table 3).

### 2.4. Proposed Putative Oxidation Mechanism

**Scheme 4:**
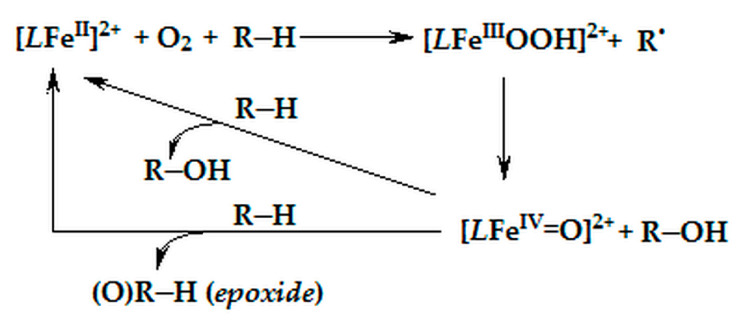
Proposed reaction for the formation of [*L*Fe^IV^=O]^2+^; *L* = Bn-tpen, R–H = cyclohexene or limonene.

Based on the electrochemical data presented in this article, it can be assumed that the [(Bn-tpen)Fe^IV^=O]^2+^ adduct is an active species in cyclohexene and limonene oxidation processes. The species is formed when the catalyst [(Bn-tpen)Fe^II^]^2+^, dioxygen, and the substrate are combined in the reaction mixture. The direct interaction of the iron(IV)-oxo complex with the substrate can lead to the production of alcohol or epoxide [12,39,49] (Scheme 4). Mechanistic details of the formation of alcohol and epoxide have been described [50,51]. However, the formation of ketone cannot be explained by this simple mechanism. 

**Scheme 5:**
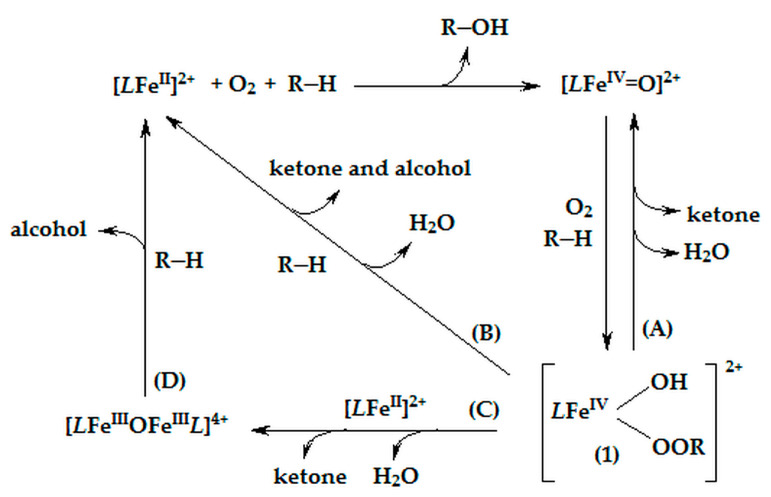
Tentative route of iron(IV)-oxo species transformations in the oxidation of R–H (cyclohexene or limonene) by dioxygen; *L* = Bn-tpen.

In previous reports [1,36,52] we postulated the formation of the adduct (**1**) (Scheme 5) as the starting point of the catalytic cycle. This adduct can decompose to form ketone and the iron(IV)-oxo species (path A); combine with another substrate molecule, giving ketone and alcohol (path B); and react with the catalyst, leading to ketone and diiron(III) μ-oxo species (path C). The diiron adduct can be reduced back to the catalyst by the organic substrate (path D), which is probably a slow process responsible for the initiation of product formation after the induction period. The rationale for the possible formation of the adduct (**1**) has been given in a previous paper [1]. Briefly, a pentadentate ligand in an iron(IV)-oxo complex can free a coordinate site of iron for a solvent molecule [32,33], which is then replaced by a dioxygen molecule. 

## 3. Materials and Methods

### 3.1. Equipment

The products of the performed reactions were identified by gas chromatography using a Hewlett-Packard (series 4890A) (Palo Alto, CA, USA) gas chromatograph with an HP-1 capillary column (cross-linked methyl-silicone gum phase, 30 m × 0.53 mm i.d.). Cyclic voltammetric measurements were performed on Princeton Applied Research (PAR, Oak Ridge, TN, USA) Model 273A and Metrohm Autolab (Utrecht, The Netherlands) model PGSTAT 302 N potentiostats. The structure of the compounds obtained as a result of the syntheses was confirmed by ^1^H NMR investigations, which were carried out in CD_3_CN at 25 °C, using an NMR 500 MHz Bruker (Billerica, MA, USA) AvanceTM spectrometer.

### 3.2. Chemicals and Reagents 

The chemicals used for syntheses or catalytic tests were of the highest purity available and were not additionally purified. Argon (grade 5.0, high-purity) from Linde (Kraków, Poland) was used to deaerate the solutions. Iron(II) perchlorate (Fe(ClO_4_)_2_^.^6H_2_O) and iron(III) perchlorate (Fe(ClO_4_)_3_) were obtained from GRF Chemicals. Acetonitrile (≥99.9%, HPLC grade) was used as a solvent, and biphenyl (PhPh, ≥99%) served as a standard in GC analysis. The organic substrates were cyclohexene (≥99%) and (R)-(+)-limonene (97%). The chemicals for the preparation of standard curves for the analyzed products, namely, cyclohexene oxide (98%), 2-cyclohexen-1-ol (95%), 2-cyclohexen-1-one (95%) or (+)-limonene oxide (97%), (R)-(–)-carvone (98%), (–)-carveol (97%), (S)-(–)-perillyl alcohol (96%), and (S)-(–)-perillaldehyde (92%), were purchased from Aldrich. The 2-picolyl chloride hydrochloride (98%) and *N*-benzylethylenediamine (97%) used for the ligand synthesis and the iodobenzene (98%) used for PhIO preparation were also delivered by Aldrich. Peracetic acid (39%) and perchloric acid (70%) were delivered by Fluka. Magnesium sulfate anhydrous (99%) and dichloromethane (99.5%) were purchased from POCH (Gliwice, Poland) and sodium carbonate (99.5%) from ChemPur (Piekary Śląskie, Poland).

PhIO was obtained according to the procedure described in [53,54]. The Bn-tpen ligand and its complex with iron(II) were synthesized using the Schlenk line to provide an inert atmosphere when necessary, according to the procedure from the literature [55,56,57]. ^1^H NMR of Bn-tpen (500 MHz, CD_3_CN, 25 °C) showed the following: δ (ppm) = 2.66 (4H, m), 3.55 (2H, s), 3.64 (2H, s), 3.69 (4H, s), 7.15 (3H, m), 7.19–7.32 (5H, m), 7.46 (3H, t), 7.60 (3H, t), 8.43 (2H, m). The given values are consistent with the spectrum reported in the literature [57]. ^1^H NMR of the complex of iron(II) with Bn-tpen is characterized by a chemical shift of the ligand signals to higher values δ.

### 3.3. Methods

Oxidation of organic substrates: The selected catalyst concentration was dissolved in deaerated acetonitrile (O_2_, 0 atm). This solution was stirred for 10 min, then saturated with air (O_2_, 0.2 atm) or oxygen (O_2_, 1 atm) depending on the reaction conditions, and the substrate (usually 1 M) was added. Excess substrate was used to minimize the oxidation of the resulting products and protect the complex against oxidative degradation. The total volume of the solution was 2.5 cm^3^, and the reaction was carried out in a vial with a total volume of 25 cm^3^. The reactions, with constant stirring and ensuring a constant concentration of the oxidant, were usually carried out for 24 h at ambient temperature (23 ± 1 °C). Volumes of 0.2 μL were withdrawn from the reaction mixture to monitor the reaction progress using gas chromatography (GC). Biphenyl (at a concentration of 5 mM for the oxidation of cyclohexene or 10 mM for limonene) was used as an internal standard. The collected product concentration results are average values from 3 independent experiments.

Electrochemical analysis: Cyclic voltammograms were recorded in a 2 cm^3^ electrochemical cell with a 3-electrode system: the working electrode, which was glassy carbon, 1 mm diameter, in PEEK (Cypress Systems, Division of ESA, Inc., Tokyo, Japan); the auxiliary electrode, which was a Pt wire; and the reference, which was an Ag/AgCl wire in an aqueous Me_4_NCl solution with a potential of 0.00 V vs. SCE [58]. The reference electrode was placed inside a Luggin capillary that was in a Pyrex tube with Vycor. Before each measurement, the working electrode was polished using Buehler Micropolish Alumina Gamma 3B and a Buehler Microcloth polishing cloth, then rinsed with deionized water and dried.

## 4. Conclusions

In acetonitrile, the [(Bn-tpen)Fe^II^]^2+^ complex activates dioxygen for alkene oxidation. However, the process is not selective. Cyclohexene is oxidized mainly to 2-cyclohexen-1-one and 2-cyclohexen-1-ol, and the amount of ketone formed is approximately two times higher than that of alcohol, especially for low concentrations of catalyst. Cyclohexene oxide is produced in smaller quantities. In the experimental conditions applied, the main oxidation products of limonene oxidation are limonene oxide, carvone, and carveol, which are formed in a molar ratio of roughly 3:2:1. Other possible oxidation products, perillaldehyde and perillyl alcohol, are formed in trace amounts. The system is slightly less reactive than the analog in which the [(N4Py)Fe^II^]^2+^ complex is used as a catalyst [1].

The voltammetric investigations suggested that the simultaneous interaction of the catalyst, substrate, and dioxygen induces the formation of the [(Bn-tpen)Fe^IV^=O]^2+^ adduct (among other iron–dioxygen adducts), which is a reactive species that generate the catalytic cycle. The possible interaction of the reactive species with the basic form of the catalyst (the [(Bn-tpen)Fe^II^]^2+^ complex) is responsible for the existence of the induction period observed in the concentration profiles for cyclohexene oxidation.

The presented investigations indicate that there is only a small difference in the results obtained in preparative and voltammetric measurements depending on whether a synthesized or in situ-prepared catalyst was used. 

## Figures and Tables

**Table 1:** A list of papers that present the physicochemical properties of iron complexes with the Bn-tpen and N4Py ligands determined by different analytical methods.

Complexes	Analytical Methods *L*–Bn-tpen	Analytical Methods *L*–N4Py
[*L*Fe^II^]^2+^	UV–Vis [11], NMR [12], CV [12]	UV–Vis [13,14,15], NMR [12,14,16], EPR [16], EXAFS [10], CV [1,12]
[*L*Fe^IV^=O]^2+^	UV–Vis [10,17,18,19,20], EXAFS [10], NMR [21], Mössbauer [10,17,20], ESI-MS [17,19], CV [19,22]	UV–Vis [10,17,18,19,20,23], X-ray [10,21], EXAFS [10], NMR [21], Mössbauer [17,20], CV [1,19,22], ESI-MS [17]
[*L*Fe^III^–OOH]^2+^	UV–Vis [11,12,24], EPR [12,25,26,27], ESI-MS [25], Mössbauer [26,27]	UV–Vis [12,13,14,16,27], EPR [12,13,14,16,27], EXAFS [27], Mössbauer [26,27]

*L*—ligand, UV–Vis—Ultraviolet–Visible Spectroscopy, NMR—Nuclear Magnetic Resonance Spectroscopy, CV—Cyclic Voltammetry, EPR—Electron Paramagnetic Resonance Spectroscopy*,* EXAFS—Extended X-Ray Absorption Fine Structure Spectroscopy, ESI-MS—Electrospray Ionization Mass Spectrometry.

**Table 2:** Oxidation of cyclohexene with dioxygen (p_O2_ = 1 atm) and air (p_O2_ = 0.2 atm) in MeCN, catalyzed by [(Bn-tpen)Fe^II^]^2+^. Reaction time 24 h.

Catalyst, mM	Substrate, M	O_2_, atm	Ketone ^a^, mM	Ketone ^b^, mM	Alcohol ^a^, mM	Alcohol ^b^, mM	Epoxide ^a^, mM	Epoxide ^b^, mM	TON ^a^	TON ^b^
0.5	1	1	116	113	46	35	12	14	348	324
0.5	1	0.2	110	118	48	44	10	15	336	354
1 ^c^	1	1	120	138	50	41	11	18	181	197
1 ^d^	1	0.2	115	115	49	51	8	7	172	173
2.5	1	1	96	111	34	20	4	12	54	57
2.5	1	0.2	80	98	20	37	7	4	43	56
5	1	1	93	84	38	16	4	3	27	21
5	1	0.2	72	65	13	23	3	2	18	18
7.5	1	1	76	48	28	15	3	1	14	9
7.5	1	0.2	62	42	20	9	2	1	11	7
10	1	1	72	23	20	6	2	1	9	3
10	1	0.2	47	21	6	5	1	1	5	3
1	0.5	1	51	43	7	8	5	6	63	57
1	0.5	0.2	38	42	12	10	5	6	55	58
1 ^c^	1	1	120	138	50	41	11	18	181	197
1 ^d^	1	0.2	115	115	49	51	8	7	172	173
1	1.5	1	182	144	47	43	25	23	254	210
1	1.5	0.2	145	143	44	54	26	20	215	217
1	2	1	218	174	94	70	30	28	342	272
1	2	0.2	196	170	138	56	22	21	356	247

^a^—synthesized [(Bn-tpen)Fe^II^]^2+^; ^b^—[(Bn-tpen)Fe^II^]^2+^ formed in situ; ^c,d^—data from the same experiments, respectively; TON—turnover number—product molecules per catalyst molecule.

**Table 3:** Oxidation of limonene with dioxygen (p_O2_ = 1 atm) and air (p_O2_ = 0.2 atm) in MeCN, catalyzed by [(Bn-tpen)Fe^II^]^2+^ formed in situ. Reaction time 24 h.

Catalyst, mM	Substrate, M	O_2_, atm	Limonene Oxide, mM	Carvone, mM	Carveol, mM	Perillaldehyde, mM	Perillyl Alcohol, mM	TON ^a^
0.5	1	1	69	41	18	0	2	260
0.5	1	0.2	55	32	20	0	0	214
1 ^b^	1	1	62	38	15	2	2	119
1 ^c^	1	0.2	59	36	20	3	1	119
2.5	1	1	56	37	16	2	4	46
2.5	1	0.2	54	32	20	2	3	44
5	1	1	50	35	16	1	1	21
5	1	0.2	44	28	20	0	0	18
7.5	1	1	56	30	15	1	2	14
7.5	1	0.2	43	24	19	0	0	11
10	1	1	59	33	16	2	3	11
10	1	0.2	47	27	19	0	1	9
1	0.5	1	34	17	7	0	1	59
1	0.5	0.2	34	16	9	0	0	59
1 ^b^	1	1	62	38	15	2	2	119
1 ^c^	1	0.2	59	36	20	3	1	119
1	1.5	1	69	50	30	1	3	153
1	1.5	0.2	62	38	26	2	4	132
1	2	1	80	48	24	3	3	158
1	2	0.2	71	36	32	1	2	142

^a^—TON (turnover number—product molecules per catalyst molecule), ^b,c^—data from the same experiments, respectively.

## Data Availability

Data are provided in the article and Supplementary Materials.

## Supplementary Materials

The following supporting information can be downloaded at: https://www.mdpi.com/article/10.3390/molecules29163755/s1, Figure S1: Product concentrations over time for the oxidation of 1 M cyclohexene by air (p_O2_ = 0.2 atm) in the presence of 10 mM cyclohexen-1-ol catalyzed by 1 mM synthesized [(Bn-tpen)Fe^II^]^2+^ in MeCN; Figure S2: The amounts of products formed in the oxidation of 1 M cyclohexene with dioxygen (p_O2_ = 1.0 atm) catalyzed by 1 mM synthesized [(Bn-tpen)Fe^II^]^2+^ in MeCN after 3 h of reaction. Analysis of products was performed (a) without triphenylphosphine and (b) with 50 mM triphenylphosphine added to the sample before analysis; Figure S3: The amounts of products formed in the oxidation of 1 M limonene with dioxygen (p_O2_ = 1.0 atm) catalyzed by 1 mM synthesized [(Bn-tpen)Fe^II^]^2+^ in MeCN after 3 h of reaction. Analysis of products was performed (a) without triphenylphosphine and (b) with 50 mM triphenylphosphine added to the sample before analysis; Figure S4: Cyclic voltammogram of 5 mM synthesized [(Bn-tpen)Fe^II^]^2+^ in MeCN containing 0.1 M (*t*-Bu)_4_NClO_4_ as a supporting electrolyte. Scan rate 0.1 V/s, GCE (0.008 cm^2^), SCE vs. NHE +0.242 V, anodic scan; Figure S5: Cyclic voltammogram of 5 mM synthesized [(Bn-tpen)Fe^II^]^2+^ in MeCN with 0.1 M (*t*-Bu)_4_NClO_4_. Scan rate 0.1 V/s, GCE (0.008 cm^2^), SCE *vs.* NHE +0.242 V; Figure S6: Dependence of I on ν^1/2^ registered for 5 mM synthesized [(Bn-tpen)Fe^II^]^2+^ in MeCN [0.1 M (*t*-Bu)_4_NClO_4_] for anodic peaks at potentials of (**a**) +1.0 V and (**b**) +1.5 V; Figure S7: Dependence of I on ν^1/2^ registered for the mixture of 5 mM synthesized [(Bn-tpen)Fe^II^]^2+^ and 10 mM PhIO in MeCN [0.1 M (*t*-Bu)_4_NClO_4_], GCE (0.008 cm^2^), SCE *vs.* NHE +0.242 V.
