# Supplementary material for: The [(Bn-tpen)FeII]2+ Complex as a Catalyst for the Oxidation of Cyclohexene and Limonene with Dioxygen
Source: Molecules. 2024 Aug 8;29(16):3755. doi: 10.3390/molecules29163755 (PMC11357577; doi:10.3390/molecules29163755)
Supplement: Supplementary file 1 [file molecules-29-03755-s001.zip › molecules-3067017-supplementary.pdf]

Supplementary Materials

The [(Bn-tpen)FeII]2+ Complex as a Catalyst for the Oxidation of Cyclohexene and Limonene with Dioxygen

Katarzyna Rydel-Ciszek * and Andrzej Sobkowiak *

Department of Physical Chemistry, Faculty of Chemistry, Rzeszów University of Technology, Al. Powstańców Warszawy 6, 35-959 Rzeszów, Poland
* Correspondence: kasiar@prz.edu.pl (K.R.-C.); asobkow@prz.edu.pl (A.S.)

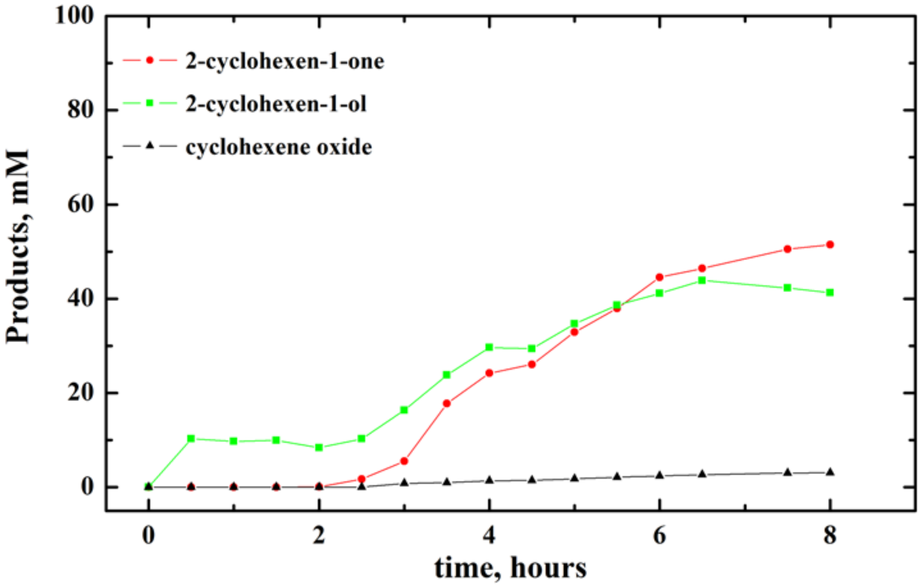

Figure S1. Product concentrations over time for the oxidation of 1 M cyclohexene by air (p_{O2} = 0.2 atm) in the presence of 10 mM cyclohexen-1-ol catalyzed by 1 mM synthesized $[(Bn\text{-}tpen)Fe^{II}]^{2+}$ in MeCN.

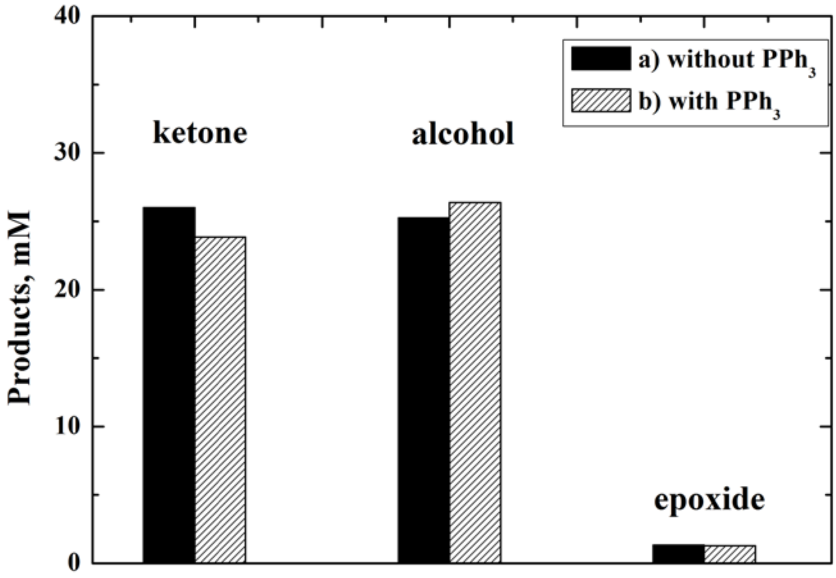

Figure S2. The amounts of products formed in the oxidation of 1 M cyclohexene with dioxygen (p_{O2} = 1.0 atm) catalyzed by 1 mM synthesized $[(Bn\text{-}tpen)Fe^{II}]^{2+}$ in MeCN after 3 h of reaction. Analysis of products was performed a) without triphenylphosphine, b) with 50 mM triphenylphosphine added to the sample before analysis.

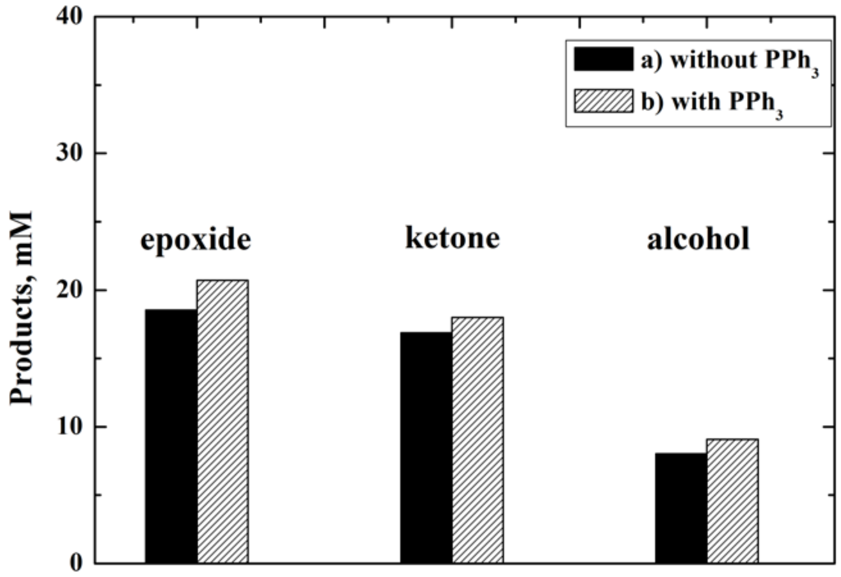

Figure S3. The amounts of products formed in the oxidation of 1 M limonene with dioxygen (p_{O2} = 1.0 atm) catalyzed by 1 mM synthesized $[(Bn\text{-}tpen)Fe^{II}]^{2+}$ in MeCN after 3 h of reaction. Analysis of products was performed a) without triphenylphosphine, b) with 50 mM triphenylphosphine added to the sample before analysis.

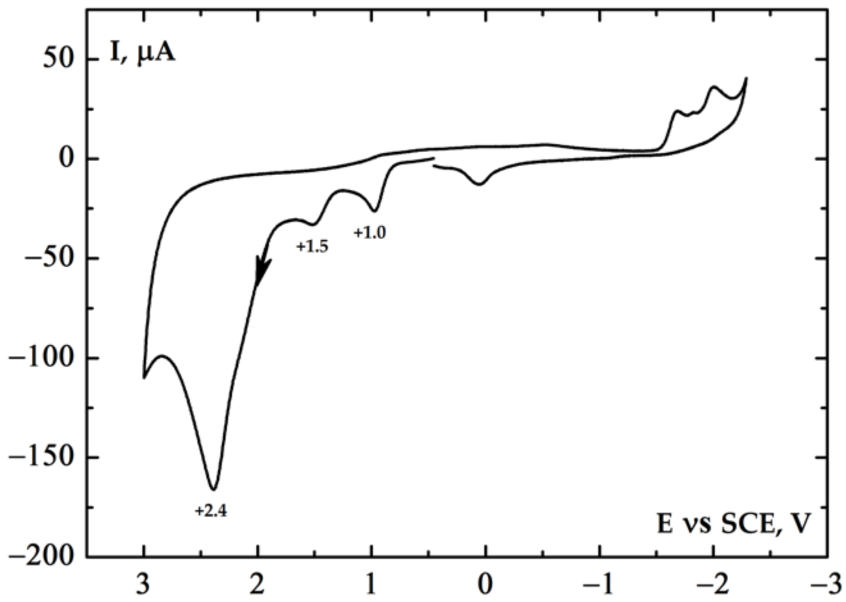

Figure S4. Cyclic voltammogram of 5 mM synthesized $[(Bn\text{-}tpen)Fe^{II}]^{2+}$ in MeCN containing 0.1 M $(t\text{-}Bu)_4NClO_4$ as a supporting electrolyte. Scan rate, 0.1 V/s, GCE (0.008 cm^2), SCE *vs.* NHE +0.242 V, the anodic scan.

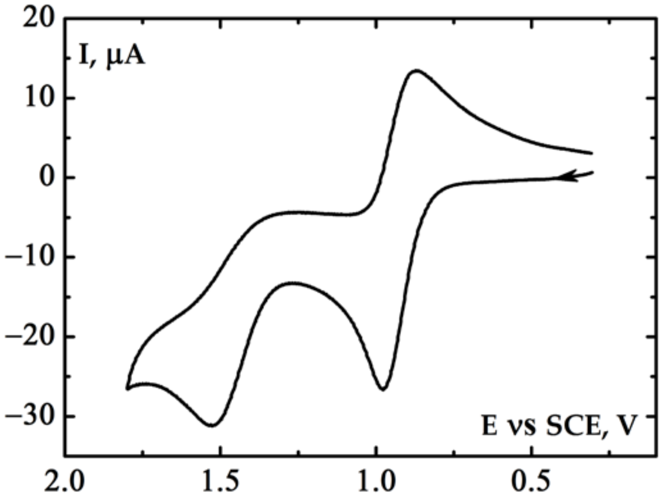

Figure S5. Cyclic voltammogram of 5 mM synthesized $[(Bn\text{-}tpen)Fe^{II}]^{2+}$ in MeCN with 0.1 M $(t\text{-}Bu)_4NClO_4$. Scan rate, 0.1 V/s, GCE (0.008 cm^2), SCE *vs.* NHE +0.242 V.

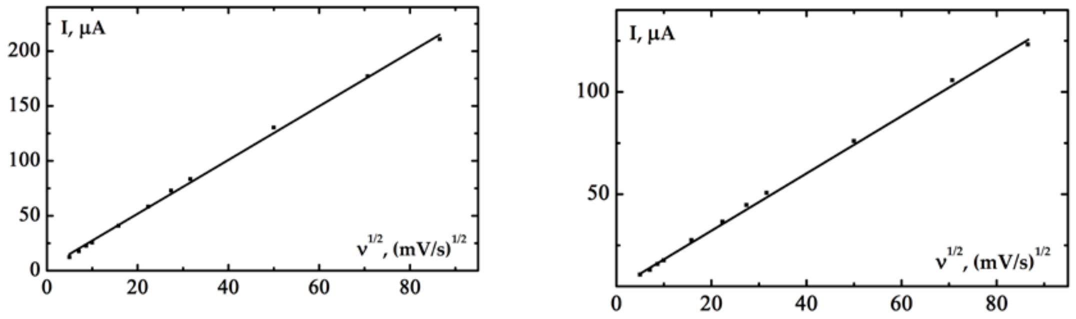

(a) **(b)**

Figure S6. Dependence of I on $\nu^{1/2}$ registered for 5 mM synthesized $[(Bn\text{-}tpen)Fe^{II}]^{2+}$ in MeCN [0.1 M $(t\text{-}Bu)_4NClO_4$] for anodic peaks at potentials **(a)** +1.0 V, **(b)** +1.5 V.

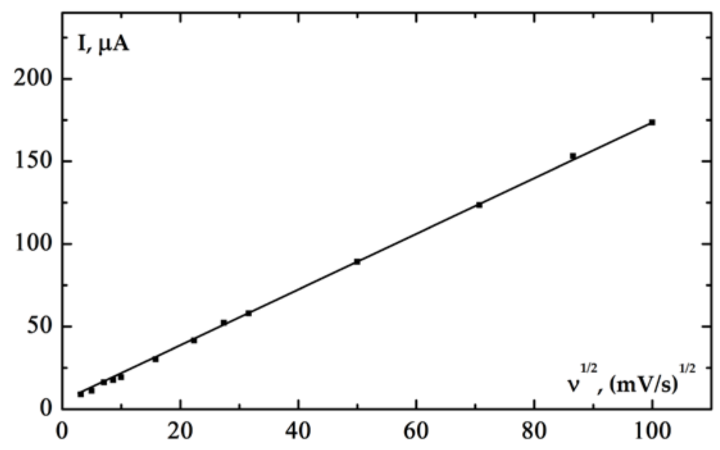

Figure S7. Dependence of I on $\nu^{1/2}$ registered for the mixture of 5 mM synthesized [(Bn-tpen)Fe^{II}]$^{2+}$ and 10 mM PhIO in MeCN [0.1 M (*t*-Bu)$_4$NClO$_4$], GCE (0.008 cm^2), SCE *vs.* NHE +0.242 V.
